# S100A6 Regulates nucleus pulposus cell apoptosis via Wnt/β-catenin signaling pathway: an in vitro and in vivo study

**DOI:** 10.1186/s10020-024-00853-4

**Published:** 2024-06-14

**Authors:** Fengguang Yang, Yanni Duan, Yanhu Li, Daxue Zhu, Zhaoheng Wang, Zhangbin Luo, Yizhi Zhang, Guangzhi Zhang, Xuegang He, Xuewen Kang

**Affiliations:** 1 Department of Orthopedics, The Second Hospital of Lanzhou University, 82 Cuiying Men, Lanzhou, Gansu Province 730030 China; 2 The Second Clinical Medical College, Lanzhou University, Lanzhou, China; 3 Orthopaedics Key Laboratory of Gansu Province, The Second Hospital of Lanzhou University, Lanzhou, 730030 China

**Keywords:** S100A6, Intervertebral disc degeneration, Apoptosis, Wnt/β-catenin signaling pathway

## Abstract

**Background:**

Intervertebral disc degeneration (IDD) is a common musculoskeletal degenerative disease, which often leads to low back pain and even disability, resulting in loss of labor ability and decreased quality of life. Although many progresses have been made in the current research, the underlying mechanism of IDD remains unclear. The apoptosis of nucleus pulposus (NP) cells (NPCs) is an important pathological mechanism in intervertebral disc degeneration (IDD). This study evaluated the relationship between S100A6 and NPCs and its underlying mechanism.

**Methods:**

Mass spectrometry, bioinformatics, and quantitative real-time polymerase chain reaction (qRT-PCR) analyses were used to screen and verify hub genes for IDD in human IVD specimens with different degeneration degrees. Western blotting, immunohistochemistry (IHC), and/or immunofluorescence (IF) were used to detect the expression level of S100A6 in human NP tissues and NPCs. The apoptotic phenotype of NPCs and Wnt/β-catenin signaling pathway were evaluated using flow cytometry, western blotting, and IF. S100A6 was overexpressed or knocked down in NPCs to determine its impact on apoptosis and Wnt/β-catenin signaling pathway activity. Moreover, we used the XAV-939 to inhibit and SKL2001 to activate the Wnt/β-catenin signaling pathway. The therapeutic effect of S100A6 inhibition on IDD was also evaluated.

**Results:**

S100A6 expression increased in IDD. In vitro, increased S100A6 expression promoted apoptosis in interleukin (IL)-1β-induced NPCs. In contrast, the inhibition of S100A6 expression partially alleviated the progression of annulus fibrosus (AF) puncture-induced IDD in rats. Mechanistic studies revealed that S100A6 regulates NPC apoptosis via Wnt/β-catenin signaling pathway.

**Conclusions:**

This study showed that S100A6 expression increased during IDD and promoted NPCs apoptosis by regulating the Wnt/β-catenin signaling pathway, suggesting that S100A6 is a promising new therapeutic target for IDD.

**Supplementary Information:**

The online version contains supplementary material available at 10.1186/s10020-024-00853-4.

## Introduction

Intervertebral disc degeneration (IDD) is a common musculoskeletal degenerative disease and the primary cause of lower back pain, leading to the loss of labor ability and reduced quality of life in patients (Global 2016, Livshits et al. 2011; Teraguchi et al. 2014). In the United States, up to 25% of people suffer from lower back and neck pain (Martin et al. 2008). In contrast, approximately 80% of people worldwide suffer from lower back pain at some point in their life (Global 2017, Borenstein 2013). Worldwide, over 600 million patients are estimated to have lower back pain. Therefore, the management of these patients has resulted in enormous social and economic burden (Katz 2006; March et al. 2014; Martin et al. 2008). IDD is related to many factors, such as age, smoking, infection, biomechanical abnormalities, and malnutrition (Roberts et al. 2006; Urban and Roberts 2003). However, the molecular mechanisms underlying IDD remain unclear.

The intervertebral disc (IVD) comprises three parts: the annulus fibrosus (AF), nucleus pulposus (NP), and cartilaginous endplate (CEP). Fibrocartilage tissue connects to the vertebral bodies IVD plays a key role in absorbing shocks and distributing mechanical loads along the spine during the bearing physical movement (Setton and Chen 2004; Zhou et al. 2014). IDD is considered the result of anabolic and catabolic imbalances accompanied by the apoptosis of NPCs and degradation of major components of the extracellular matrix, such as type II collagen (collagen II) and aggrecan (Galbusera et al. 2014). To identify the underlying molecular mechanism of IDD, we collected surgical specimens of degenerative IVDs (grade IV) and nondegenerative IVDs (grade II) from patients with scoliosis according to Pfirrmann grading (Pfirrmann et al. 2001) and performed mass spectrometry analysis for the experimental and control groups. Combined with the IDD-related genes in the Comparative Toxicogenomics Database (CTD) and GeneCards, and the IDD-related gene expression profile dataset GSE23130 downloaded from the Gene Expression Omnibus (GEO) database, bioinformatics analysis and validation were performed. S100A6 expression is increased in degenerative NP tissues, which may be related to IDD.

S100A6, also known as calcyclin, is a low-molecular-weight Ca^2+^-binding protein that belongs to the S100 protein family. The S100 protein family contains more than 20 members with most coding genes, including S100A6, clustered on human chromosome 1q21(Marenholz et al. 2004). S100A6 exists in various mammalian tissues and cells and is closely related to many neoplastic diseases, such as lung cancer (Wang et al. 2021b), colorectal cancer (Wang et al. 2017), pancreatic cancer (Zihao et al. 2013), melanoma (Mitamura et al. 2014), and liver cancer (Song et al. 2020). Moreover, S100A6 is also involved in non-neoplastic diseases, such as Alzheimer’s disease (Boom et al. 2004), amyotrophic lateral sclerosis (Hoyaux et al. 2000), epilepsy (Jurewicz et al. 2013), acute lymphoblastic leukemia (Pui et al. 2002), osteoarthritis (Lourido et al. 2021), and endometriosis (Gomes et al. 2018). S100A6 has several molecular functions related to apoptosis and differentiation (Gross et al. 2014; Tsoporis et al. 2008), and tumor invasion, migration, and proliferation (Komatsu et al. 2002). Studies have shown that S100A6 participates in the progression of osteoarthritis (Lourido et al. 2017). However, to our knowledge, no in vitro or in vivo studies have evaluated the relationship between S100A6 and apoptosis in IDD.

Wnt/β-catenin is a highly conserved branch of Wnt signaling, in which the C-terminus of β-catenin acts as a binding factor for various complexes and promotes β-catenin-mediated transcription upon the entry into the nucleus. In contrast, the phosphorylation of the N-terminus promotes β-catenin degradation (Schaefer et al. 2018). The activation of the Wnt signaling pathway is associated with cell proliferation, apoptosis, migration, angiogenesis, and other processes (Vallée et al. 2021). In recent years, several studies have shown that Wnt/β-catenin signaling pathway plays an important role in IDD (Wang et al. 2021a, 2022; Zhan et al. 2019). Kondo N et al. (Kondo et al. 2011) found that Wnt/β-catenin plays a key role in IVD development through Wnt/β-catenin reporter (TOPGAL) mice. Cai Z et al. (Cai et al. 2023) found that the activation of Wnt/β-catenin signaling pathway decreases the viability of NPCs and increases their apoptosis rate. Interestingly, S100A6 expression was synergistic with β-catenin in eutopic endometrial stromal cells (Liu et al. 2015; Zhang et al. 2017). However, whether S100A6 is associated with apoptosis induced by Wnt/β-catenin activation in NPCs is still unclear.

This study, investigated the role and mechanism of action of S100A6 in IDD. The expression of S100A6 in human NP increases with IDD severity. S100A6 mediates the apoptosis of NPCs via Wnt/β-catenin signaling pathway. An AF puncture-induced rat model of IDD confirmed the protective effects of S100A6 inhibition in vivo. The results of this study improve our understanding of the mechanism of action of S100A6 in IDD and provide reference data for developing IDD-targeted therapies.

## Materials and methods

### Human NP tissue collection

The experimental protocol involved in this study was approved by the Ethics Committee of The Second Hospital of Lanzhou University. Procedures involving human subjects followed the guidelines of the Declaration of Helsinki, and disc tissues were obtained with written informed consent from all patients. The degeneration group (grade IV) included 21 patients (11 males, 10 females; age 52–75 years, mean 65.3 years) who underwent discectomy and spinal fusion. The normal group (grade II) included 19 patients (9 males, 10 females; age, 12–17 years, mean 14.6 years) who underwent scoliosis correction surgery. All patients received surgical treatment at The Second Hospital of Lanzhou University, and those with a history of tumors, tuberculosis and other infectious diseases were excluded before surgery.

### Label-free proteomic profiling

The samples were then removed from the frozen state and placed on ice. An appropriate amount of protein lysate (8 M urea, 1% SDS) containing protease inhibitors was used to inhibit protease activity. The mixture was ultrasonicated for 2 min at a low temperature, followed by splitting for 30 min. After centrifugation at 12,000 g at 4 °C for 30 min, the concentration of protein supernatant was determined by bicinchoninic acid (BCA) method by BCA Protein Assay Kit (Pierce, Thermo, USA). Proteins were quantified according to the manufacturer’s instructions.

Label-free proteomic profiling of the protein extracts was performed by Shanghai Majorbio Co., Ltd. In protein samples of 100 µg, TEAB (triethylammonium bicarbonate buffer) was added; therefore, the final concentration of TEAB was 100 mM. Then TCEP (tris [2-carboxyethyl] phosphine) was added to the final concentration of 10 mM and reacted for 60 min at 37 °C. Next, IAM (Iodoacetamide) was added to the final concentration of 40 mM and reacted for 40 min at room temperature under dark conditions. A certain percentage (acetone: sample v/v = 6:1) of precooled acetone was added to each sample and allowed to settle for 4 h at − 20 °C. After centrifugation for 20 min at 10,000 g, the sediment was collected and 100 µL 100 mM TEAB solution was added to dissolve it. Finally, the mixture was digested with Trypsin overnight at 37 °C added at 1:50 trypsin-to-protein mass ratio. The peptides were vacuum-dried and resuspended in 0.1% TFA. The samples were desalted using HLB and vacuum dried. Peptide concentrations were determined using a peptide quantification kit (Thermo, Cat.23,275).

The original files were imported from the mass spectrometry system into ProteomeDiscovererTM Software 2.2 a software system for database analysis. The identified proteins contained at least one specific peptide segment. The searched data were uploaded to a Major Cloud platform (Cloud. majorbio. com) for analysis. We used the protein abundance information obtained by searching the database for differential protein statistical analysis. Student’s t-test was used to calculate the P-value of the difference between samples. *P* < 0.05, and fold change ≥ 1.2 were used to identify differentially expressed proteins (DEPs) (Shi et al. 2018; Zhang et al. 2019).

### Preprocessing of the IDD gene expression profile dataset and DEGs screening

We downloaded the GSE23130 (www.ncbi.nlm.nih.gov/geo/query/acc.cgi?acc=GSE23130) gene expression profile dataset associated with IDD from the GEO database. (https://www.ncbi.nlm.nih.gov/) on the GPL1352 [U133_X3P] Affymetrix Human X3P Array platform. The expression profile dataset was used to analyze the expression of genes in IVDs with different degrees of degeneration. We used GEO2R (https://www.ncbi.nlm.nih.gov/) to perform differential gene analysis, *P* < 0.05, and fold change ≥ 1.2 were used to identify DEGs. Genes related to IDD were obtained from the Comparative Toxicogenomics Database (https://ctdbase.org/) and GeneCards (https://www.genecards.org/). Venn analysis was performed using the DEGs obtained from the GSE23130 dataset. The obtained results were then analyzed with the top 100 DEPs by mass spectrometry to obtain hub genes of IDD, and subsequent verification was conducted.

### Culture and treatment of human NPCs

Human NPCs were purchased from Shanghai Yaji Biotechnology Co. Ltd. (article number: 0028a, China). Cultivate Dulbecco’s modified Eagle’s medium cultured Nutrient Mixture F-12 (DMEM/F-12) (Gibco, Thermo Fisher Scientific) containing 10% fetal bovine serum (Biosharp, Hefei, China) in a 37

°C incubator containing 5% CO_2_. The culture medium 2–3 times per week. Under an optical microscope, when the cell density reaches 80–90%, the cells were digested with 0.25% trypsin ethylenediaminetetraacetic acid (Gibco, Thermo Fisher Scientific) and passaged in a 1:2 ratio. NPCs were added to six-well plates at a density of 1 × 10^5^ cells/well. According to previously described, NPCs were pretreated (or not) with LV-shS100A6, LV-S100A6, or LV-NC viruses and cultured for 24 h with or without the addition of interleukin (IL)-1β (10 ng/mL, PeproTech, USA) (Chen et al. 2018) and the Wnt/β-catenin inhibitor XAV-939 (10 µM, Selleck, China) or activator SKL2001 (40 µM Selleck, China), respectively (Li et al. 2017; Xu et al. 2016). When the cell fusion rate was 70–80%, and then collected.

### RNA extraction and quantitative real-time polymerase chain reaction (qRT-PCR)

Total ribonucleic acid (RNA) from human NP tissue and NPCs was extracted according to Trizol (Takara, bio Inc.) manufacturer’s instructions. Reverse transcription of total RNA was performed using the PrimeScript RT Master Mix (Takara Bio Inc.) according to the manufacturer’s instructions. The primers used for qRT-PCR are listed in Table 1. The expression levels of MMP13, COL6A2, FLOT1, S100A6, and SOD1 were detected using the SYBR PrimeScript RT-PCR Kit (Takara Bio Inc.). The target mRNA expression levels were normalized to those of GAPDH. The relative expression levels were calculated using the 2^−ΔΔCT^ method.

**Table 1:** Sequences of primers used for quantitative real-time PCR

Gene	Forward (5–3′)	Reverse (5–3′)
MMP13	ACTGAGAGGCTCCGAGAAATG	GAACCCCGCATCTTGGCTT
COL6A2	GACACCATCAACCGCATCAT	AGGCAGCTCACCTTGTAGCAC
FLOT1	GCCCTGCATCCAACAGATCC	AATGCCAGTGACTGAGATGGG
S100A6	GAACAAGGACCAGGAGGTG	TTGAGGGCTTCATTGTAGAT
SOD1	GGTGGGCCAAAGGATGAAGAG	CCACAAGCCAAACGACTTCC
GAPDH	CAGCCTCAAGATCATCAGCA	ATGATGTTCTGGAGAGCCCC

### Western blotting

RIPA lysis buffer (Beyotime, Shanghai, China) was used to extract proteins from the NP tissues and NPCs. The Bradford protein assay kit (Thermo Fisher Scientific) was used to determine the protein concentration. Equal amounts of protein were separated by 10% sodium dodecyl sulfate-polyacrylamide gel electrophoresis (SDS-PAGE) and transferred to polyvinylidene fluoride (PVDF) membranes (Millipore, Billerica, MA, USA). Quickblock™ Western blocking solution was blocked at room temperature for 15 min, and then in the corresponding antibody concentration overnight at 4 ℃: Collagen II (1:1000, Abclonal), MMP-13 (1:2000, Proteintech), S100A6 (1:1000, Abcam), p16 (1:2000, Immunoway), Caspase-9 (1:1000, Proteintech), Caspase 3 (1:1000, Cell Signaling Technology), Cleaved caspase 3 (1:2000, Immunoway), Bax (1:1000, Proteintech), BCL-2 (1:1000, Proteintech), β-catenin (1:1000, Abcam), Wnt3a (1:1000, Biorbyt), and GAPDH (1:1000, Proteintech). The membrane was then washed thrice with TBST (Beyotime, Shanghai, China) for 5 min each, followed by incubation with horseradish peroxidase-conjugated goat anti-rabbit antibody (1:5000, Proteintech) or horseradish peroxidase-conjugated goat anti-mouse antibody (1:5000, Proteintech) for 1 h at room temperature. Protein signals were detected using a hypersensitive luminescence solution (New Cell Molecular Biotech, Suzhou, China) and ChemiDoc XRS imaging system (Bio-Rad). ImageJ software was used for the gray-scale analysis. All experiments were performed thrice.

### Lentivirus transfection

When the density of NPCs in the cell culture bottle was approximately 30%, the lentivirus (GeneChem) was transfected with a multiple infection of 100. The medium was changed 48 h after transfection. The expression of S100A6 was quantified using qRT-PCR and western blotting.

### Apoptosis assays

Apoptosis was detected using an Annexin V-APC/7-AAD apoptosis kit (MultiSciences Biotech) according to the manufacturer’s instructions. Briefly, the cells were centrifuged and washed with precooled PBS and collected 1–5 × 10^5^ cells were collected. Then used 500 µL 1 × binding buffer to resuspend cells. Next, 5 µL annexin V-APC and 10 µL 7-AAD were added to each tube. After gentle vortex mixing, the mixture was incubated in the dark at room temperature for 5 min, and then online analysis was performed. The apoptosis rate was determined using a flow cytometer (FC500; Beckman Coulter, Brea, CA, USA).

### Immunohistochemical (IHC)

The rats were sacrificed 8 weeks after the operation, and the tail vertebrae were obtained. After fixation with 4% paraformaldehyde, decalcification, dehydration, and embedding, 5 μm thick sections were prepared (human disc specimens were exempted from decalcification). After antigen retrieval and endogenous enzyme clearance, these sections were incubated with primary antibodies (S100A6, Collagen II, MMP13, Cleaved caspase 3 and β-catenin) at 4 °C overnight. Then, these sections were incubated with goat anti-rabbit or anti-mouse immunoglobulin (Ig)G-horseradish peroxidase HRP (1:500; ZSGB Bio, China) at 37 ° C for 30 min. Finally, 3’,3-diaminobenzidine tetrahydrochloride solution was added, and the nuclei were stained with hematoxylin and observed under light microscope.

### Hematoxylin-eosin (HE) and Safranin-O/fast green staining

To evaluate the degree of degeneration of human and rat IVDs, the tissues fixation with 4% paraformaldehyde, decalcification, dehydration, and embedding, 5 μm thick sections were prepared (human disc specimens were exempted from decalcification). HE staining kit (Solarbio, Beijing, China) and modified Safranin -O/fast green stain kit (Solarbio, Beijing, China) were used according to the manufacturer’s instructions for HE and Safranin-O/fast green staining.

### Immunofluorescence staining (IF)

Slides were placed in six-well plates and NPCs were seeded onto them. After the corresponding treatment, the medium was removed and the cells were washed three times with precooled PBS for 3 min each time. The cells were fixed with 4% paraformaldehyde for 15 min and washed thrice with PBS for 3 min each. Next, the cells were permeabilized with 0.5% Triton X-100 at room temperature for 20 min, and the slides were washed thrice with PBS for 3 min each. It was blocked with 10% goat serum for 1 h at room temperature and incubated with primary antibody at 4 ℃ overnight. After washing thrice with PBS for 3 min each time, the cells were incubated with Alexa fluor 488/594-goat anti-rabbit lgG at 37 ℃ for 1 h. Finally, 4′, 6-diamidino-2-phenylindole (DAPI, Invitrogen) was added dropwise to label the nuclei for 10 min. A fluorescence microscope was used to capture the images (Olympus Inc., Tokyo, Japan). ImageJ software was used to measure fluorescence intensity.

### Animal experiments

Previous studies have shown that the AF puncture-induced rat model of IDD is mature IDD model (Chen et al. 2018). According to the experimental arrangement, we randomly divided SD rats into Sham, IDD, IDD + LV-NC and IDD + LV-shS100A6 groups. The rats were sedated with isoflurane and anesthetized with pentobarbital (40 mg/kg via intraperitoneal injection). The skin was cut under sterile conditions to expose the co7/8 IVD and a syringe needle (21G) was used to pierce the NP with the needle direction parallel to the CEP. The needle was rotated 360° in the NP and stayed for 1 min. Microinjector syringes were used for lentivirus injection to reduce injection volume error. The experimental operator was blinded to the study protocol.

### Magnetic resonance imaging (MRI)

To evaluate the effect of LV-shS100A6 on the treatment of IDD, we used a 3.0 T MR scanner (Siemens, Erlangen, Germany) 8 weeks after surgery. The T2-weighted MRI sequence has certain advantages in monitoring the water content of NP tissue (Jin et al. 2018). Serial T2-weighted sagittal slices were obtained using the following settings: a fast-spin echo sequence with a time-to-repetition of 5,400 ms and, time-to-echo of 920 ms, 320 (h) 9256 (v) matrix, a field-of-view of 260°, and four excitations. The section thickness was 2 mm with a gap of 0 mm. The tail vertebrae of the rats were analyzed according to the classification method described by Pfirrmann et al. (Pfirrmann et al. 2001).

### Data analysis

The results are expressed as the mean ± standard deviation, and the results were based on at least three independent replicate experiments. Statistical analyses were performed using the GraphPad Prism software (ver. 8.0). Student’s t-test or one-way analysis of variance (ANOVA) was used to compare differences between groups. *P* < 0.05 was considered statistically significant.

## Results

### Screening of hub genes in IDD

**Fig. 1:**
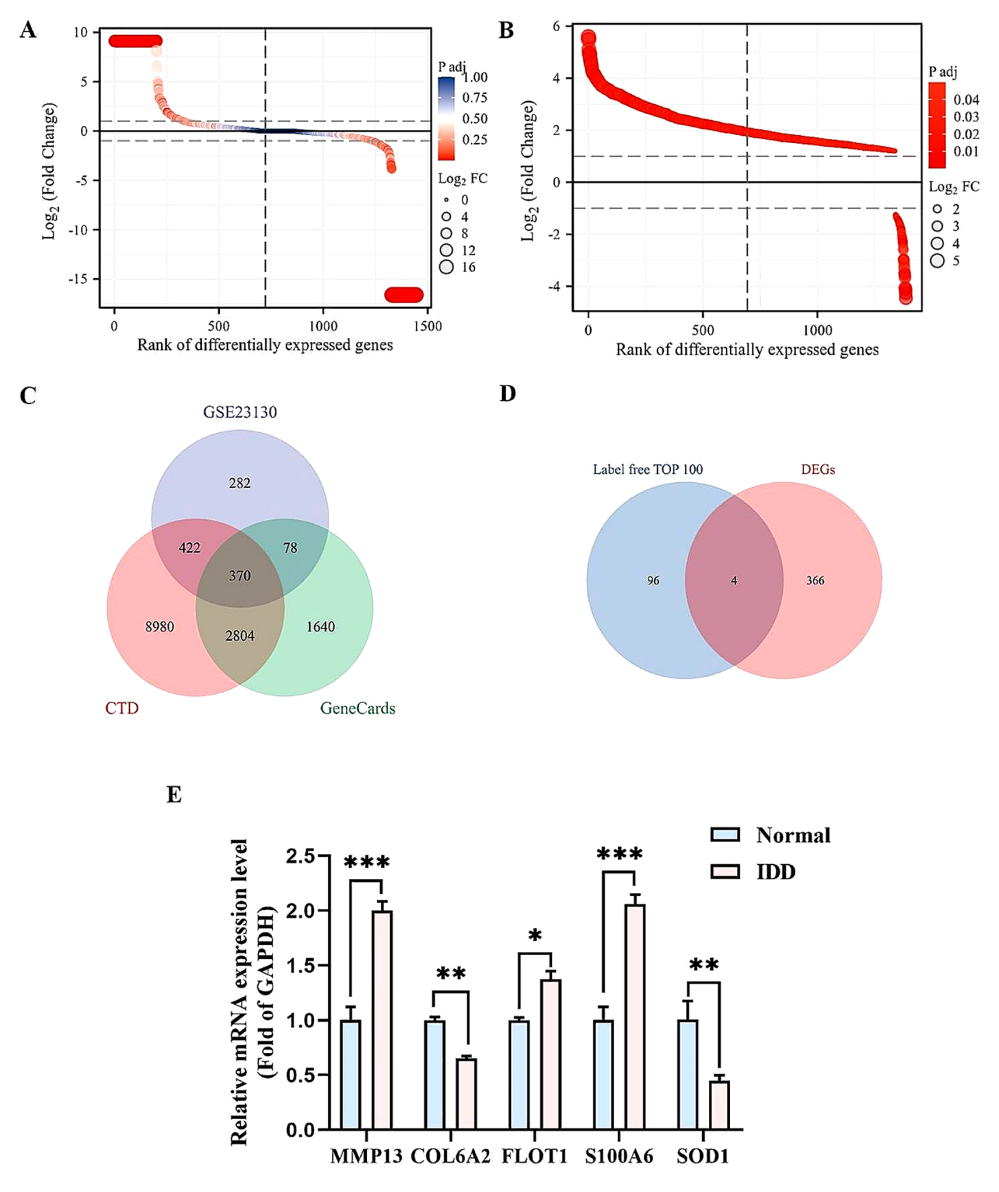
Screening of hub genes in IDD. (**A**)Mass spectrometry analysis revealed the differentially expressed genes. The colors represent P-values and circles represent Log2FC. (**B**) Differentially expressed genes in IDD according to the GSE23130 dataset analysis. The colors represent P-values and circles represent Log2FC. (**C**) Venn analysis showing DEGs in GSE23130 dataset, CTD, and GeneCards (370 common genes were obtained). (**D**) Venn analysis showing the TOP100 proteins of DEPs in mass spectrometry and the result of (**C**) four co-expressed hub genes (COL6A2, FLOT1, S100A6, and SOD1). (**E**) Expression levels of MMP13, COL6A2, FLOT1, S100A6, and SOD1 in human IVDs of Pfirrmann Grade II and Grade IV detected using qRT-PCR. * *P* < 0.05, ** *P* < 0.01, and *** *P* < 0.001

To explore the molecular mechanisms underlying IDD, surgical specimens of IVDs with different degrees of degeneration were collected. According to the Pfirrmann classification, patients were divided into an experimental group (*n* = 4, grade IV) and a control group (*n* = 4, grade II) for mass spectrometry analysis (label-free). Mass spectrometry results revealed 369 DEPs, of which 234 were upregulated and 135 were downregulated (Fig. 1A). To search for hub genes of IDD, we analyzed the expression profile dataset of IDD-related genes GSE23130 and found a total of 1,152 DEGs (Fig. 1B). We combined them with IDD-related genes in the CTD (12,576 genes) and Genecards (4,892 genes) to conduct Venn analysis, and obtained 370 common genes (Fig. 1C). We screened the differential proteins obtained by mass spectrometry analysis using Cytoscape software, performed Venn analysis on the top 100 proteins and the above results, and obtained four co-expressed differential genes (COL6A2, FLOT1, S100A6, and SOD1) (Fig. 1D). qRT-PCR was used to detect the expression of MMP13, COL6A2, FLOT1, S100A6, and SOD1 in Pfirrmann grade II and IV human IVD specimens (Fig. 1E). Among these, S100A6 expression was significantly different, and the literature has reported many molecular functions (Donato et al. 2017). We took S100A6 as the target gene in subsequent experiments.

### The expressions of S100A6, cleaved caspase 3, and β-catenin were increased in human degenerative NP tissues

**Fig. 2:**
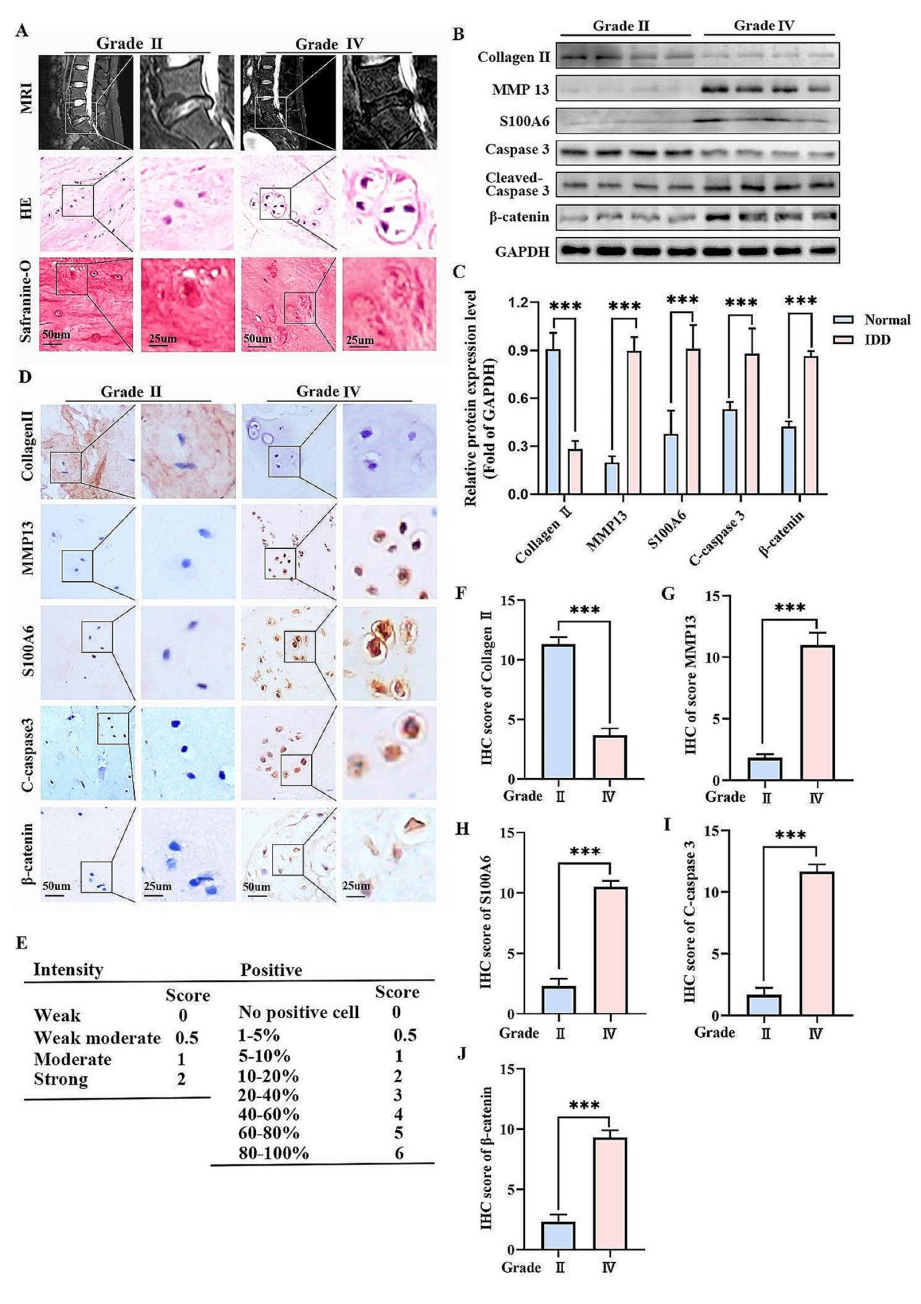
The expressions of S100A6, cleaved caspase 3, and β-catenin were increased in human degenerative NP tissues. (**A**) Human IVD MRI examination, HE, and Safranin-O/fast green staining with Pfirmmann grading Grade II and Grade IV. (**B, C**) Western blot and quantitative analysis of S100A6, cleaved caspase 3, β-catenin, MMP13, and Collagen II in Grade IV and Grade II groups. (**D-J**) IHC staining and quantitative analysis of S100A6, cleaved caspase 3, β-catenin, MMP13 and Collagen II were performed in Grade IV and Grade II groups. * *P* < 0.05, ** *P* < 0.01, and *** *P* < 0.001

S100A6 is a Ca^2+^-binding protein expressed in a limited number of cell types in normal adult tissues and several tumor cell types (Duan et al. 2014; Li et al. 2016). S100A6 regulates a variety of cellular functions, such as proliferation, apoptosis, migration, and cellular responses to different stress factors (Donato et al. 2017; Li et al. 2014). We collected IVD specimens with different degrees of degeneration by preoperative MRI examination with reference to the Pfirrmann grading, and HE and Safranin-O/fast green staining revealed different degrees of degeneration (Fig. 2A). Then, we performed western blotting experiments and found that S100A6, cleaved caspase 3, β-catenin, and MMP13 in the degeneration group (grade IV) was higher than that in the normal group (grade II), while the expression of collagen II was the opposite (Fig. 2B, C). The same results were obtained by IHC (Fig. 2D-J). This indicated that apoptosis of NPCs increased during IDD, while S100A6 and β-catenin expression increased.

### The expression of S100A6, cleaved caspase 3, and β-catenin increased in the AF puncture-induced rat IDD model

**Fig. 3:**
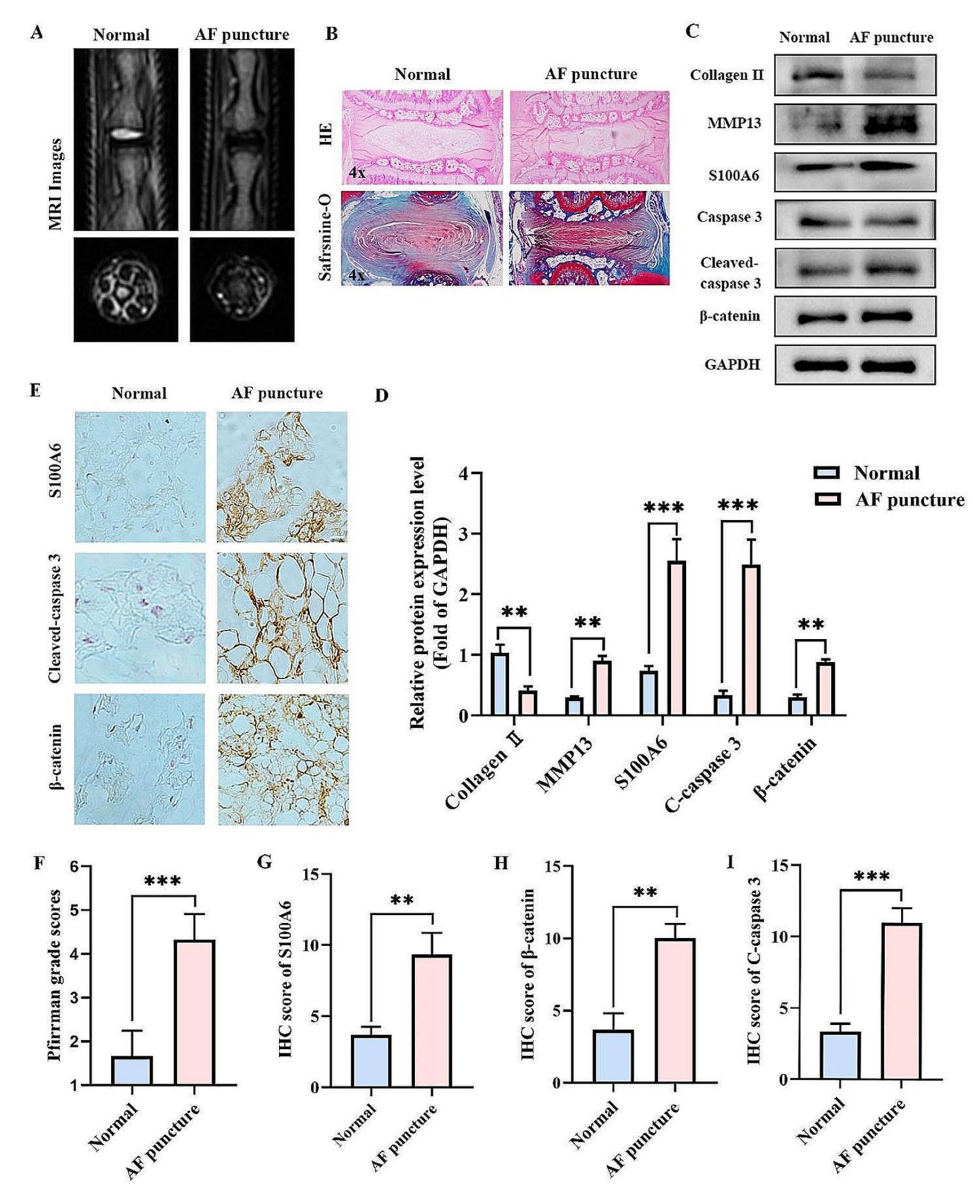
The expressions of S100A6, cleaved caspase 3, and β-catenin increased in the AF puncture-induced rat IDD model. (**A, F**) MRI examination and Pfirrmann score were performed 8 weeks after surgery in the AF puncture and normal groups. (**B**) HE and Safranin-O/fast green staining at 8 weeks after surgery in the AF puncture and normal groups. (**C, D**) Western blot and quantitative analysis of S100A6, cleaved caspase 3, β-catenin, MMP13, and Collagen II in the AF puncture and normal groups. (**E, G-I**) IHC staining and quantitative analysis of S100A6, cleaved caspase 3, and β-catenin in the AF puncture and normal groups. * *P* < 0.05, ** *P* < 0.01, and *** *P* < 0.001

To further evaluate the expression levels of S100A6, cleaved caspase 3 and β-catenin, we used the AF puncture-induced rat IDD model. Eight weeks after the operation, MRI of the rat tail vertebrae was performed. The T2 images showed that the water content of the IVD in the AF-puncture group was lower and the Pfirrmann score was higher than that in the normal group (Fig. 3A, F). HE and Safranin-O/fast green staining showed that the NP collapsed, the height of the IVD decreased and order of AF was disordered during AF puncture (Fig. 3B). Subsequently, we performed western blotting experiments and found S100A6, cleaved caspase 3, β-catenin, and MMP13 in AF puncture group was higher than that in normal group, while the expression of collagen II was opposite (Fig. 3C, D). This indicates that the apoptosis of NPCs is increased, and the expressions of S100A6 and β-catenin also increased in the AF puncture-induced rat IDD model.

### The expression of S100A6 and β-catenin, and the apoptosis rate of human NPCs were concentration-dependent with IL-1β

**Fig. 4:**
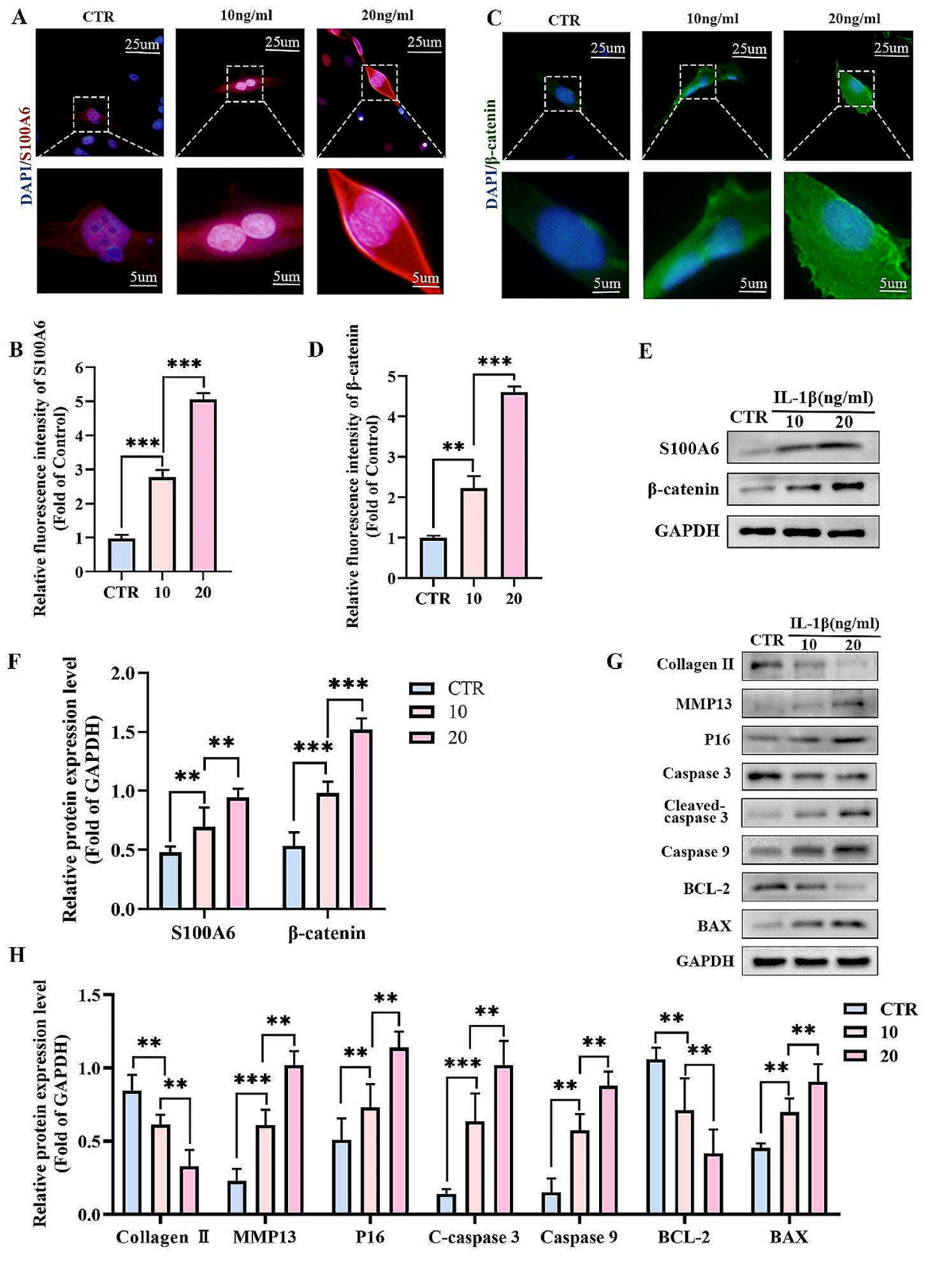
The expression of S100A6 and β-catenin and the apoptosis rate of human NPCs were concentration-dependent with IL-1β. (**A-D**) IF staining and quantitative analysis of S100A6 and β-catenin in human NPCs treated with different concentrations of IL-1β. (**E-H**) Western blot and quantitative analysis of S100A6, β-catenin, P16, cleaved caspase 3, caspase 9, BAX, and MMP13 in human NPCs treated with different concentrations of IL-1β. * *P* < 0.05, ** *P* < 0.01, and *** *P* < 0.001

In the process of IDD, many inflammatory factors such as IL-1β, IL-6, IL-7, tumor necrosis factor-α accumulation in the IVD, causing the imbalance of catabolism and anabolism, leading to the structural changes of the IVD and promoting the progress of IDD (Risbud and Shapiro 2014). Previous studies have reported that IL-1β is involved in various pathological processes and promote IDD (Risbud and Shapiro 2014). In addition, inhibition of IL-1β can promote the synthesis of extracellular matrix, thereby inhibiting IDD (Genevay et al. 2009; Le Maitre et al. 2007). Therefore, IL-1β-induced NPC model has been used in studies of IDD (Chen et al. 2018). IF showed that the expression levels of S100A6 and β-catenin in NPCs increased with the increase of IL-1β concentrations (Fig. 4A-D). Western blott showed that the expression of S100A6, β-catenin, P16, cleaved caspase 3, caspase 9, Bax, and MMP13 gradually increased. The expression levels of collagen II and BCL-2 gradually decreased (Fig. 4E-H).

### Targeted regulation of S100A6 can affect the apoptosis and the activity of Wnt/β-catenin signaling pathway in IL-1β-induced human NPCs

**Fig. 5:**
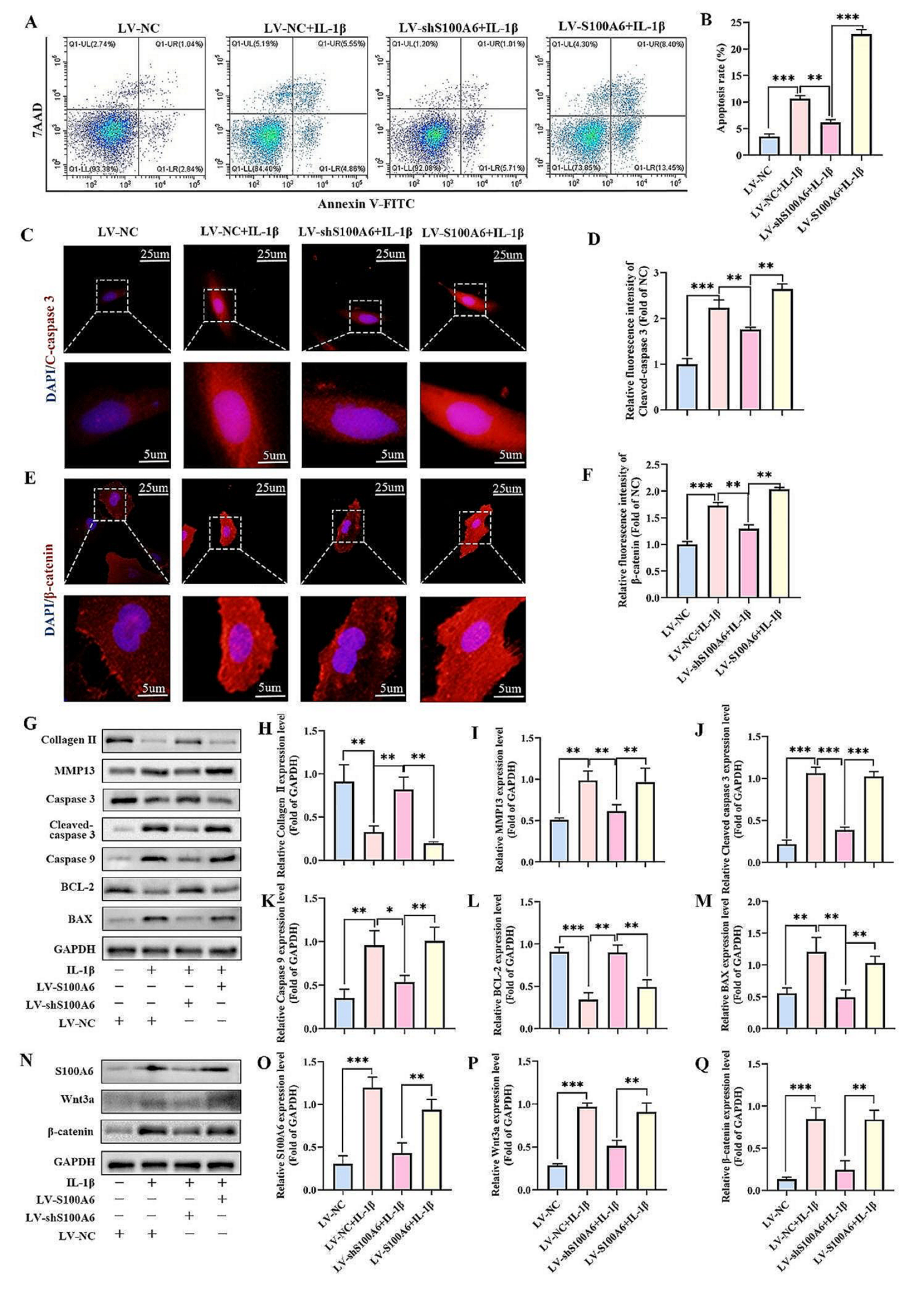
Targeted regulation of S100A6 can affect the apoptosis and the activity of Wnt/β-catenin signaling pathway in IL-1β-induced human NPCs. (**A**) The apoptosis rates of NPCs determined using flow cytometry. (**B**) Percentages of apoptotic cells. (**C-F**) IF staining and quantitative analysis of S100A6 and β-catenin. (**G-M**) Western blot and quantitative analysis of cleaved caspase 3, caspase 9, BAX, BCL-2, MMP13, and Collagen II. (**N-Q**) Western blot and quantitative analysis of Wnt3a and β-catenin. * *P* < 0.05, ** *P* < 0.01, and *** *P* < 0.001

Further to explore the relationship between S100A6 and NPCs apoptosis, we used lentivirus to achieve targeted S100A6 adjustment. From Fig. S1 A-B, the results of western blot showed that the S100A6-shRNA lentivirus (LV-shS100A6) and S100A6 overexpression lentivirus (LV-S100A6) could precisely knock down or upregulate the expression of S100A6. The verification results of qRT-PCR is similar to western blot (Fig. S1C). Subsequently, cell apoptosis was detected by flow cytometry (Fig. 5A, B). The apoptosis rate of NPCs after IL-1β treatment (NC + IL-1β group) was significantly higher than that in the normal group (NC group), while the apoptosis rate of in LV-shS100A6 + IL-1β group was significantly lower than that in the NC + IL-1β group. However, the apoptosis rate of LV-S100A6 + IL-1β group was significantly higher than that of the other three groups. IF showed that the expression of cleaved caspase 3 in the NC + IL-1β group was significantly higher than that in the NC group, but the expression of LV-shS100A6 + IL-1β group was lower than that in the NC + IL-1β group. However, the expression level of LV-S100A6 + IL-1β group was higher than that of LV-shS100A6 + IL-1β group (Fig. 5C, D). The expression trend of β-catenin was similar to that of cleaved caspase 3 (Fig. 5E, F).

The results of western blot showed that cleaved caspase 3, caspase 9, and MMP13 were increased in the NC + IL-1β group and the LV-S100A6 + IL-1β group, whereas their expression levels were decreased in the LV-shS100A6 group. The opposite trend was observed for collagen II and BCL-2 (Fig. 5G-M). Wnt3a and β-catenin showed the same expression trend as cleaved caspase 3. This suggests that S100A6 may regulate the apoptosis of NPCs by activating Wnt3a/β-catenin pathway.

### Inhibition of Wnt/β-catenin signaling pathway partially reversed IL-1β-induced human NPCs apoptosis caused by S100A6 overexpression

**Fig. 6:**
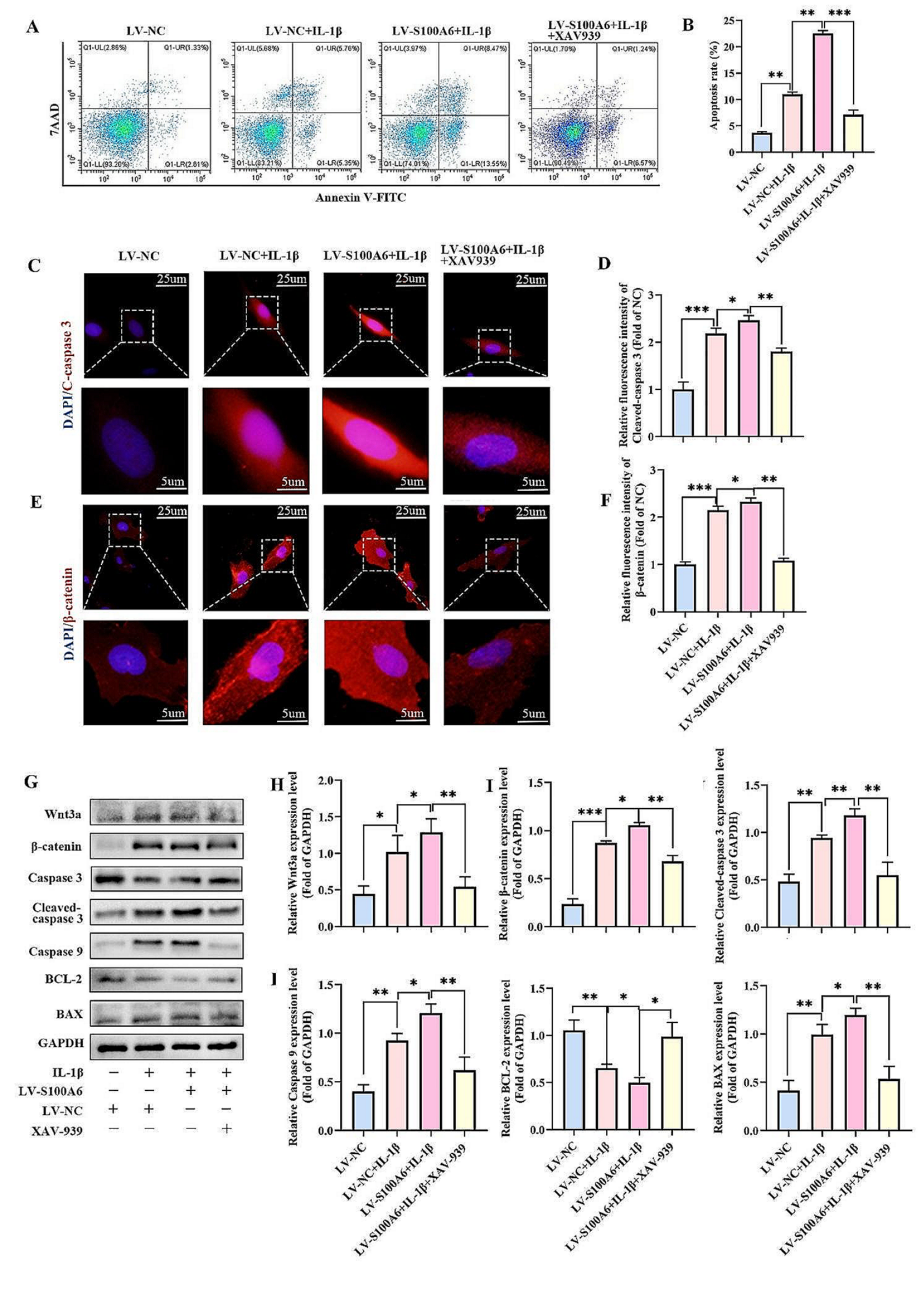
Inhibition of Wnt/β-catenin signaling pathway partially reversed IL-1β-induced human NPCs apoptosis caused by S100A6 overexpression. (**A**) The apoptosis rates of NPCs were determined using flow cytometry. (**B**) Percentages of apoptotic cells. (**C-F**) IF and quantitative analysis of S100A6 and β-catenin. (**G-M**) Western blot and quantitative analysis of cleaved caspase 3, caspase 9, BCL-2, BAX, Wnt3a, MMP13, β-catenin and Collagen II. * *P* < 0.05, ** *P* < 0.01, and *** *P* < 0.001

To further explore the role of Wnt/β-catenin signaling pathway in S100A6-mediated apoptosis of NPCs, XAV-939(a Wnt/β-catenin signaling pathway inhibitor) was added to the LV-S100A6 + IL-1β group. Western blot and IF results showed that XAV-939 inhibited the expression of Wnt3a and β-catenin proteins in the LV-S100A6 + IL-1β group (Fig. 6. G-I). Compared with the LV-NC group, the expression levels of cleaved caspase 3, caspase 9, and Bax in the NC + IL-1β group and the LV-S100A6 + IL-1β group significantly increased, and the expression level of BCL-2 was decreased. The expression levels of cleaved caspase 3, caspase 9, and Bax in LV-S100A6 + IL-1β + XAV-939 group were significantly lower than the LV-S100A6 + IL-1β group. The expression of BCL-2 in LV-S100A6 + IL-1β + XAV-939 group was higher than that in LV-S100A6 + IL-1β group (Fig. 6G, J-M). IF revealed that the expression of cleaved caspase 3 trends and western blot results were consistent (Fig. 6C, D). The results of flow cytometry also showed that compared with the LV-S100A6 + IL-1β group, the apoptosis rate of the LV-S100A6 + IL-1β + XAV-939 group was reduced (Fig. 6A, B). This indicated that the inhibition of Wnt/β-catenin signaling pathway could partially reverse the apoptosis effect of NPCs caused by S100A6 overexpression.

### Activation of Wnt/β-catenin signaling pathway could partially reverse the anti-apoptosis of IL-1β-induced human NPCs caused by S100A6 knockdown

**Fig. 7:**
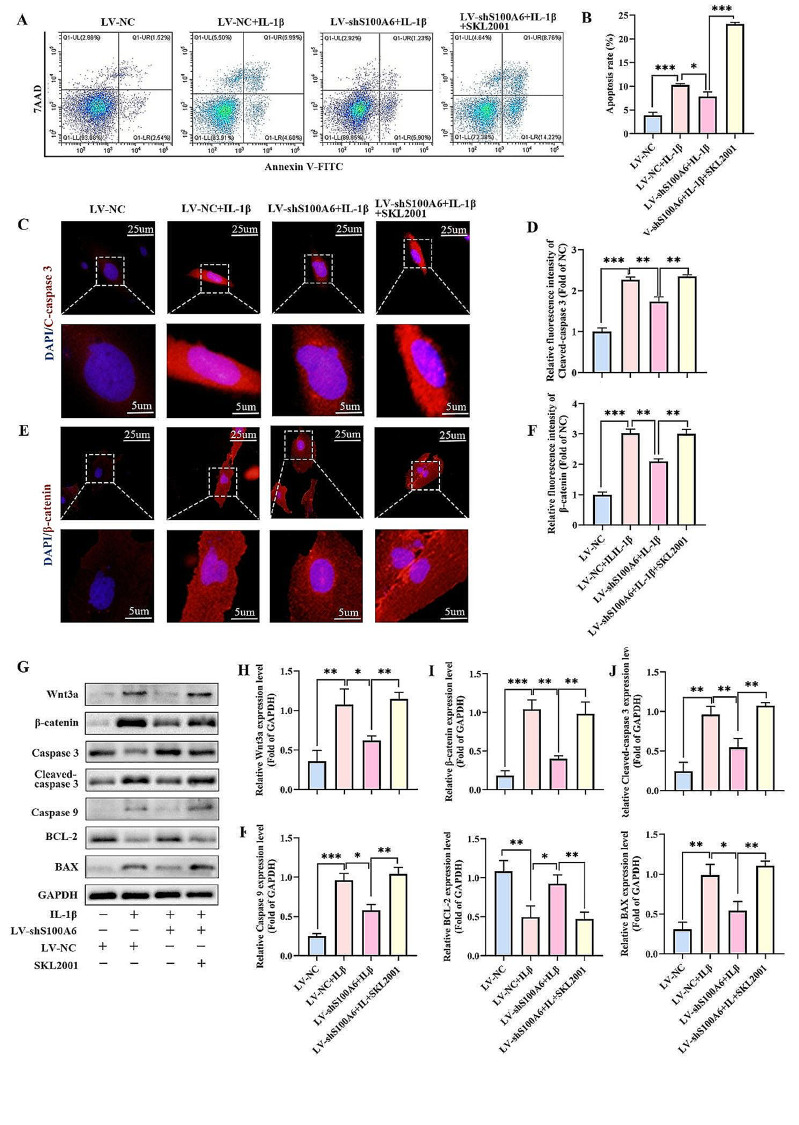
Activation of Wnt/β-catenin signaling pathway could partially reverse the anti-apoptosis of IL-1β-induced human NPCs caused by S100A6 knockdown. (**A**) The apoptosis rates of NPCs were determined using flow cytometry. (**B**) Percentages of apoptotic cells. **(C-F**) IF and quantitative analysis of S100A6 and β-catenin. (**G-M**) Western blot and quantitative analysis of cleaved caspase 3, caspase 9, BCL-2, BAX, Wnt3a, MMP13, β-catenin, and Collagen II. * *P* < 0.05, ** *P* < 0.01, and *** *P* < 0.001

To further investigate whether Wnt/β-catenin signaling activation could reverse anti-apoptotic caused by S100A6 knockdown in IL-1β-induced human NPCs. SKL2001(a Wnt/β-catenin signaling pathway activator) was added to the LV-shS100A6 + IL-1β group. Western blot and IF showed SKL2001 application increased the LV-shS100A6 + IL-1β group Wnt3a and β-catenin protein expression level (Fig. 7E-I). Compared with the LV-NC + IL-1β group, the expression levels of cleaved caspase 3, caspase 9, and Bax in the LV-shS100A6 + IL-1β group significantly decreased, and the expression level of BCL-2 increased. However, compared with the LV-shS100A6 + IL-1β group, the expression levels of cleaved caspase 3, caspase 9, and Bax in the LV-shS100A6 + IL-1β + SKL2001 group significantly increased, and the expression level of BCL-2 decreased (Fig. 7G, J-M). IF analysis showed that the expression trend of cleaved caspase 3 was consistent with the results of western blot (Fig. 7C, D). The results of flow cytometry also showed that the apoptosis rate of LV-shS100A6 + IL-1β + SKL2001 group was significantly higher than that of LV-shS100A6 + IL-1β group (Fig. 7A, B). These results suggested that Wnt/β-catenin signaling pathway activation could partially reverse anti-apoptotic caused by S100A6 knockdown in IL-1β-induced human NPCs.

### S100A6 inhibition alleviated IDD progression in vivo

**Fig. 8:**
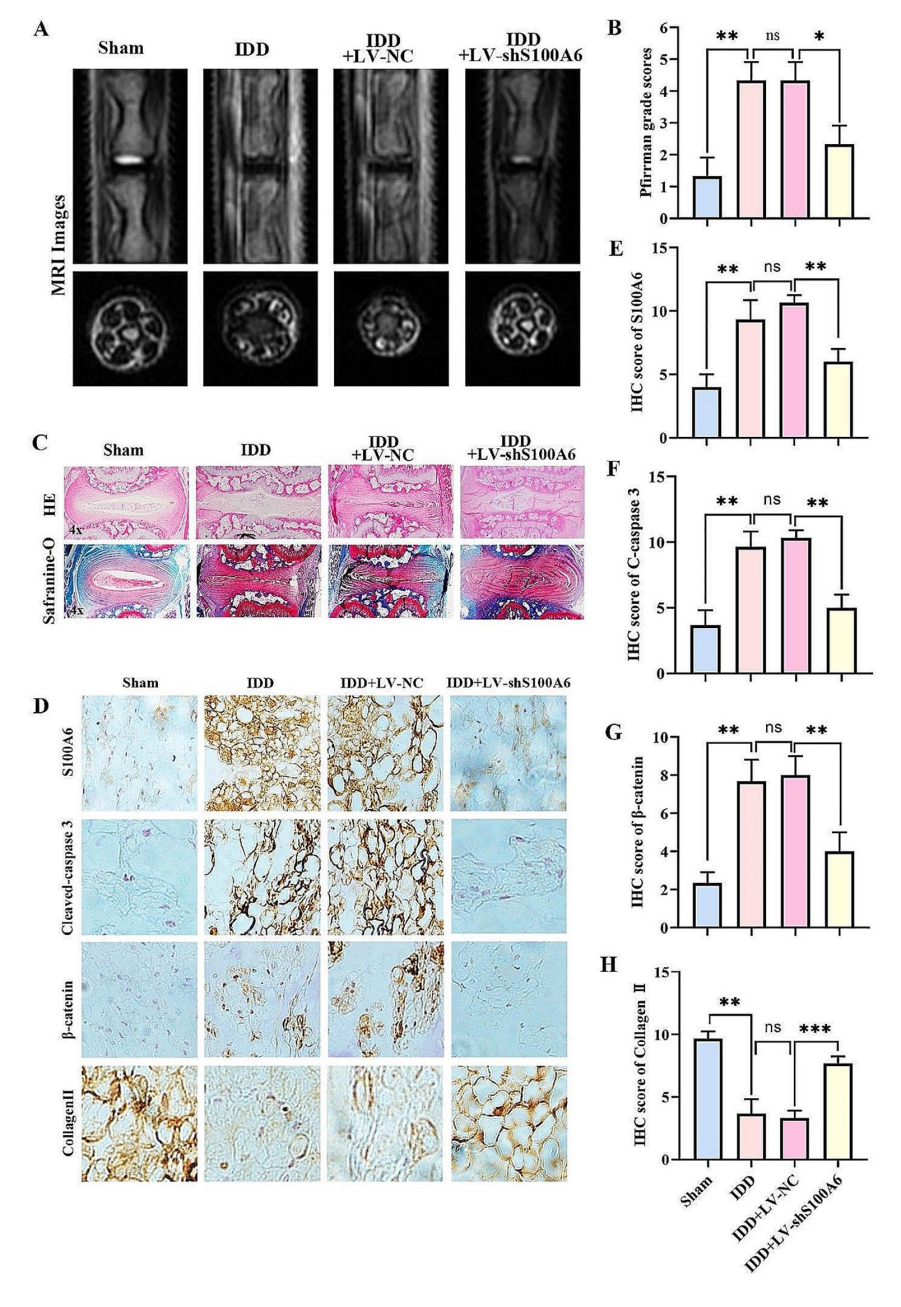
S100A6 inhibition alleviated IDD progression in vivo. (**A, B**) MRI examination and Pfirrmann score at 8 weeks after surgery. (**C**) HE and Safranin-O/fast green staining at 8 weeks after surgery. (**D-H**) IHC staining and quantitative analysis of S100A6, cleaved caspase 3, β-catenin, and Collagen II at 8 weeks after operation. * *P* < 0.05, ** *P* < 0.01, and *** *P* < 0.001

To evaluate the effect of S100A6 inhibition on IDD in vivo, we used an AF puncture-induced IDD rat model. SD rats were randomly divided into sham, IDD, IDD + LV-NC, and IDD + LV-shS100A6 groups (*n* = 24). In addition to the sham group, the rest of the group were injected physiological saline, LV-NC and LV-shS100A6. The water content of the intervertebral disc in the IDD + LV-shS100A6 group was significantly higher than those in the IDD and IDD + LV-NC groups. Correspondingly, the Pfirrmann score of the IDD + LV-shS100A6 group was significantly lower than those of the IDD and IDD + LV-NC group (Fig. 8A, B). Inhibition of S100A6 partially alleviated IDD, as shown by HE and Safranin-O/fast green staining (Fig. 8C). IHC analysis showed that S100A6 levels significantly reduced in the IDD + LV-shS100A6 group compared with those in the IDD and IDD + LV-NC groups. This confirmed the successful transfection of LV-shS100A6 via IVD injection. Importantly, IHC staining for cleaved caspase-3, β-catenin, and collagen II showed that in IDD and IDD + LV-NC groups, cleaved caspase-3 and β-catenin expression levels were increased, while the expression level of collagen II decreased significantly, indicating that NPC apoptosis and ECM catabolism increased in the process of IDD. These degenerative changes significantly decreased in the IDD + LV-shS100A6 group (Fig. 8D-H). In general, S100A6 inhibition can reduce NPC apoptosis, thereby alleviating IDD in vivo. Importantly, IHC staining for cleaved caspase-3, β-catenin, and collagen II showed that the expression levels of cleaved caspase-3 and β-catenin in IDD and IDD + LV-NC groups increased, while the expression level of collagen II decreased significantly, indicating that NPC apoptosis and ECM catabolism increased in the process of IDD. These degenerative changes significantly decreased in the IDD + LV-shS100A6 group (Fig. 8D-H). S100A6 inhibition reduced NPC apoptosis, thereby alleviating IDD progression in vivo.

## Discussion

The NP is located at the center of the IVD and is the most important bearer of biomechanical function (Jahnke and McDevitt 1988; Shapiro et al. 2012). IDD involves many pathological processes, among which apoptosis leads to a reduced number of NPCs, which is an important cause of IDD (He et al. 2021; Huang et al. 2012; Xia et al. 2019). Depletion of NPCs contributes to the initiation and acceleration of IDD (Zhong et al. 2021). In this study, we found that S100A6 regulates the apoptosis of NPCs through Wnt/β-catenin signaling pathway.

S100A6 is a Ca^2+^-binding protein belonging to the S100 protein family. Usually, S100A6 is expressed in the cytoplasm; however, S100A6 can be anchored to the plasma and nuclear membranes in a Ca^2+^ environment (Lesniak and Filipek 1996; Stradal and Gimona 1999). One S100A6 monomer binds two Ca^2+^ ions with different affinities. Where, one Ca^2+^ ion binds to an atypical EF-hand loop located at the N-terminus and the other Ca^2+^ ion binds to a typical EF-hand loop located at the C-terminus of the molecule (Filipek et al. 2008). Binding with Ca^2+^ ions can induce changes in the conformation of S100A6, exposing the hidden hydrophobic region and inducing interactions with target molecules and Ca^2+^ signal transduction (Filipek and Leśniak 2018; Sastry et al. 1998). S100A6 has various molecular functions such as apoptosis, differentiation, migration, and proliferation (Gross et al. 2014; Tsoporis et al. 2008; Yang et al. 2023). S100A6 is involved in osteoarthritis, tumors, nervous system disease, endometriosis, cardiovascular disease, and other related diseases (Yang et al. 2023). An affinity proteomic approach for detecting serum from patients with radiographic knee OA (rKOA) revealed elevated protein levels of S100A6, C3, and ITIH1. After a comparative analysis with the control group, the researchers concluded that these three serum proteins can be used as diagnostic markers for rKOA (Lourido et al. 2017). Another study showed that S100A6, C3, and ITIH1 were associated with the progression of rKOA (Lourido et al. 2021). NPCs are chondrocytes (Gan et al. 2021), with characteristics similar to those of osteoarticular chondrocytes. However, whether S100A6 is involved in IDD remains unclear, and the role of S100A6 in NPC apoptosis has not been reported. Therefore, we evaluated the expression of S100A6 in patients with IDD and found that S100A6 expression was upregulated in human degenerative NP tissues, which has also been found in cellular and rat models of IDD. In addition, the elevated expression of S100A6 was accompanied by higher levels of cleaved caspase 3, caspase 9, and Bax and lower levels of BCL-2 than that in the control group. The inhibition S100A6 expression had the opposite effect on these apoptotic markers, indicating that S100A6 positively correlates with NPC apoptosis.

Activated Wnt signaling pathway, suppressed β-catenin phosphorylation, limited degradation, and accumulated β-catenin into the nucleus play an important role in cell proliferation, apoptosis, migration, angiogenesis, and other processes (Vallée et al. 2021). Wnt/β-catenin signaling pathway plays an important role in IDD. With the progression of IDD, β-catenin positive cells also increase (Iwata et al. 2015). Cai Z et al. (Cai et al. 2023) revealed that the activation of Wnt//β-catenin signaling pathway inhibits the NPCs proliferation and promote their apoptosis, thereby accelerating the progress of IDD. Shi Z et al. (Shi et al. 2022) found that high hydrostatic pressure promoted apoptosis and inhibited the viability of human NPCs by activating Wnt/β-catenin signaling pathway. Our results are consistent with those of previous studies. We found that S100A6 overexpression activates Wnt/β-catenin signaling pathway, promoting the apoptosis of NPCs, whereas downregulating the S100A6 expression inhibits Wnt/β-catenin signaling pathway, reducing cell apoptosis. To further confirm the role of Wnt/β-catenin signaling pathway in S100A6 regulation of NPC apoptosis, we treated the S100A6-overexpressed group with XAV-939, and found that compared with the S100A6-overexpressed untreated group cell apoptosis was partially reversed. In addition, we treated the S100A6-downregulated group with SKL2001 and found that compared with the untreated S100A6-downregulated group, apoptosis increased, indicating that S100A6 regulates the apoptosis of NPCs through Wnt/β-catenin signaling pathway. It’s been well known that Ca^2+^-binding proteins modulate Hippo signaling (Sayedyahossein et al. 2023) and also β-Catenin-signaling require YAP/Hippo signaling for cell survival and tumorigenesis (Rosenbluh et al. 2012), while it has also been shown recently that Hippo signaling is involved in drug resistance (Mukhopadhyay et al. 2023). Therefore, whether S100A6 regulates Hippo signaling pathway and its biological effects are worthy of further exploration in future studies.

Although our study confirmed that elevated expression of S100A6 in IDD promotes NPC apoptosis, our study has several limitations. First, the IVD is composed of AF, CEP and NP. We only focused on the effect of S100A6 on NPCs and ignored its effects on AF and CEP, which will also be a research topic in the future. Second, our study only investigated the regulatory mechanism of S100A6 on NPCs through Wnt/β-catenin signaling pathway. However, it is unknown whether other mechanisms of action exist. Future studies should focus on the mechanism of action of S100A6 in NPCs. Third, although the AF puncture-induced rat IDD model can simulate human IDD to a certain extent, rats are rodents, and their IVDs are different from those of mammals regarding functional characteristics and biomechanical properties. Future research needs to choose an animal model closer to humans so the results can better reveal the mechanism of human IDD.

## Conclusion

**Fig. 9:**
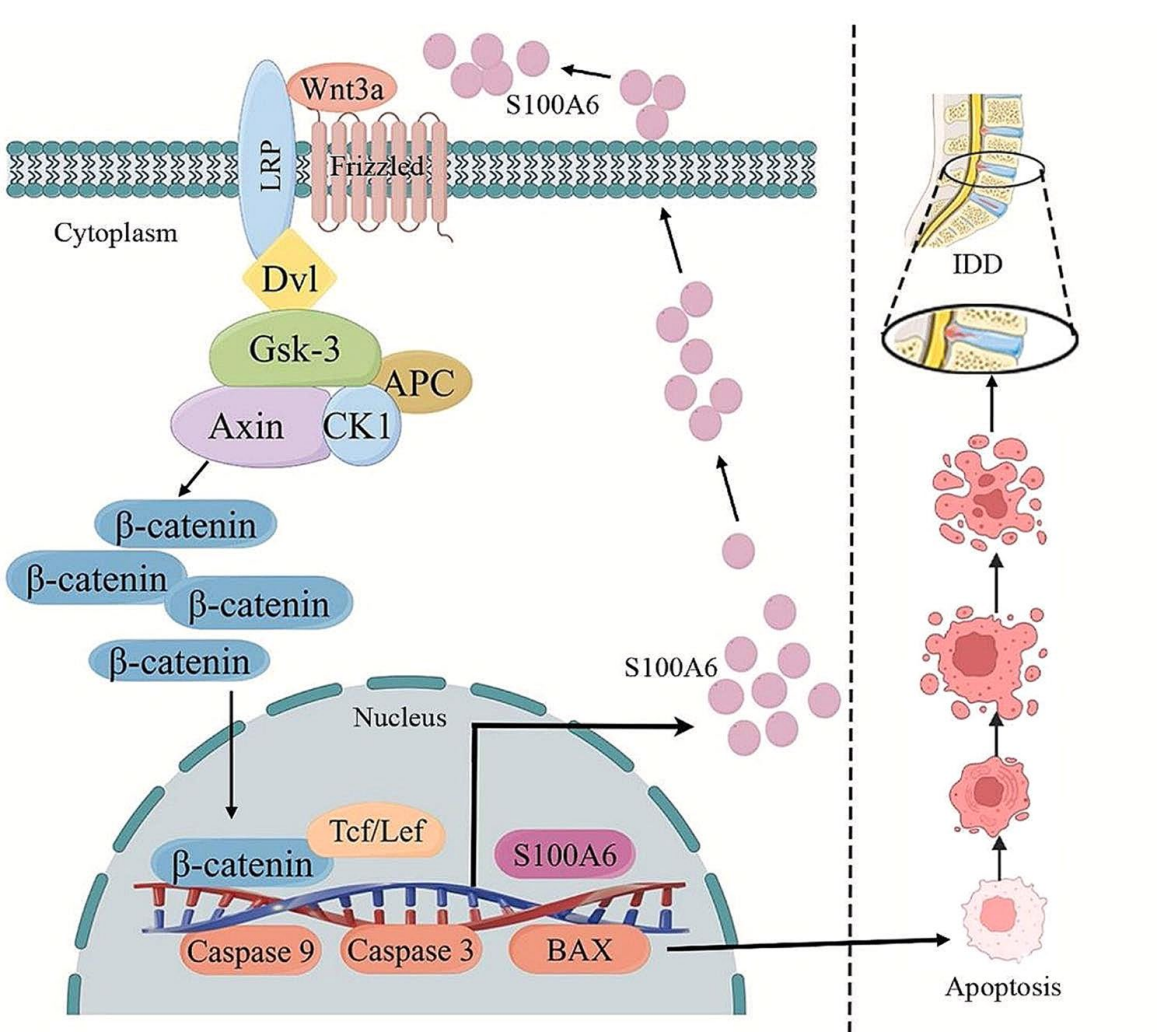
S100A6 mediates the apoptosis of human NPCs by regulating the activity of Wnt/β-catenin signaling pathway

S100A6 expression increased in degenerative human IVD tissues. In vitro, S100A6 affected the apoptosis rate of NPCs by regulating the activity of Wnt/β-catenin signaling pathway. In vivo, S100A6 inhibition partially alleviated IDD induced by AF puncture in rats (Fig. 9). Our findings suggest that S100A6 is a potential therapeutic target in IDD.

### Electronic supplementary material

Below is the link to the electronic supplementary material.

Supplementary Material 1

## Data Availability

The data that support the findings of this study are available from the corresponding authors upon reasonable request.
