# Supplementary material for: S100A6 Regulates nucleus pulposus cell apoptosis via Wnt/β-catenin signaling pathway: an in vitro and in vivo study
Source: Mol Med. 2024 Jun 14;30:87. doi: 10.1186/s10020-024-00853-4 (PMC11179208; doi:10.1186/s10020-024-00853-4)
Supplement: Supplementary file 1 — Supplementary Material 1 [file 10020_2024_853_MOESM1_ESM.docx]

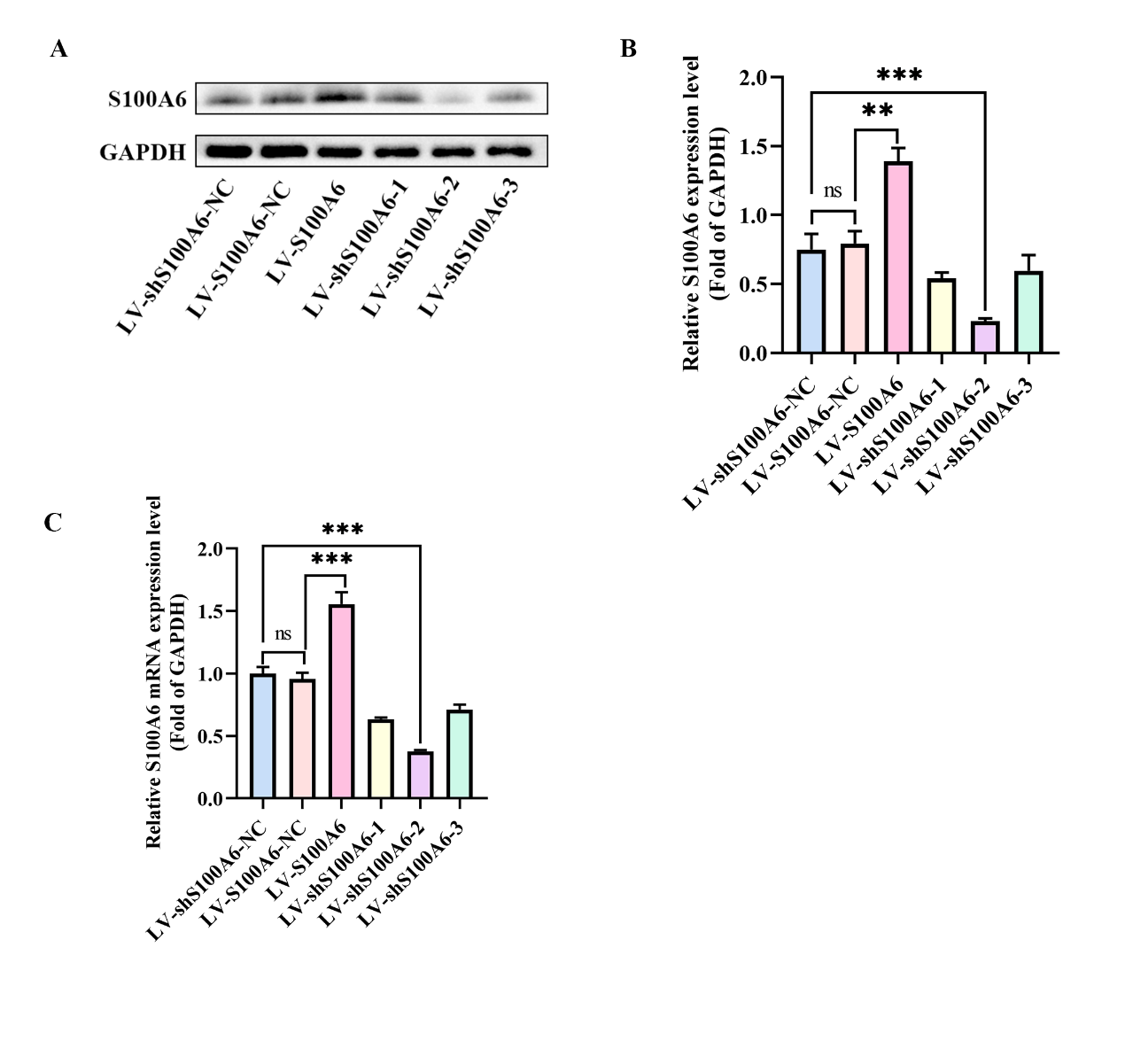


**Supplemental figure. 1** Evaluation of lentivirus transfection effect. (**A, B**) Western blot and quantitative analysis of S100A6 in each group. (**C**) qRT-PCR was used to detect the expression levels of S100A6 in each group. * *P* < 0.05, ** *P* < 0.01, and *** *P* < 0.001.
